# The Dynamics of Bird Diversity in the New World

**DOI:** 10.1093/sysbio/syaa028

**Published:** 2020-04-25

**Authors:** Antonin Machac

**Affiliations:** s1 Biodiversity Research Centre, University of British Columbia, 2212 Main Mall, Vancouver V6T 1Z4, Canada; s2 Center for Theoretical Study, Charles University, Jilska 1, 110 00 Praha 1, Czech Republic; s3 Department of Ecology, Charles University, Vinicna 7, 12844 Praha 2, Czech Republic; s4 Center for Macroecology, Evolution and Climate, University of Copenhagen, Universitetsparken 15, DK-2100 Copenhagen, Denmark

## Abstract

Three prominent explanations have been proposed to explain the dramatic differences in species richness across regions and elevations, (i) time for speciation, (ii) diversification rates, and (iii) ecological limits. But the relative importance of these explanations and, especially, their interplay and possible synthesis remain largely elusive. Integrating diversification analyses, null models, and geographic information systems, I study avian richness across regions and elevations of the New World. My results reveal that even though the three explanations are differentially important (with ecological limits playing the dominant role), each contributes uniquely to the formation of richness gradients. Further, my results reveal the likely interplay between the explanations. They indicate that ecological limits hinder the diversification process, such that the accumulation of species within a region gradually slows down over time. Yet, it does not seem to converge toward a hard ceiling on regional richness. Instead, species-rich regions show suppressed, but continued, diversification, coupled with signatures of possible competition (esp. Neotropical lowlands). Conversely, species-poor, newly-colonized regions show fast diversification and weak to no signs of competition (esp. Nearctic highlands). These results held across five families of birds, across grid cells, biomes, and elevations. Together, my findings begin to illuminate the rich, yet highly consistent, interplay of the mechanisms that together shape richness gradients in the New World, including the most species-rich biodiversity hotspots on the planet, the Andes and the Amazon. [Biogeography; community; competition; macroevolution; phylogenetics; richness gradient.]

When Humboldt traveled the New World, he was astonished by the dramatic differences in species richness across regions and elevations, from the lowlands of the tropics to the mountaintops of the temperate (Humboldt and Bonpland 1807). His observations have inspired biologists ever since (Wallace 1889; Rosenzweig 1995; Mittelbach et al. 2007). Eventually, three prominent classes of explanations for the differences in richness have emerged: (i) time for speciation (Willis 1922; Stephens and Wiens 2003), (ii) diversification rates (Dobzhansky 1950; Ricklefs 2006), and (iii) ecological limits (Simpson 1953; Rabosky 2013) (Table 1). Even though each of the explanations has been empirically supported, their synthesis remains elusive. My study attempts such synthesis by investigating the explanations side by side, with particular focus on their interplay. Specifically, I investigate richness gradients across regions and elevations within five well-known families of birds, spanning 1500+ species distributed throughout of the New World, including the Amazon and the Andes, which inspired much of the seminal research in biology, including that of Humboldt.

**Table 1.** Three prominent explanations for differences in species richness. Each explanation (time, diversification, and ecological limits) is introduced together with its diagnostic predictions regarding present-day diversification. Building on previous theory, I also test an integrative scenario that combines the effects of time, diversification, and ecological limits, while producing its own predictions.

Explanation	Rationale	Predictions	References
Time	Species-rich regions have been colonized for a long time over which they have gradually accumulated their high richness.	Present-day diversification is similar across regions.Species-rich regions host comparatively older faunas than species-poor regions.	Willis (1922), Ricklefs and Schluter (1994), Stephens and Wiens (2003)
Diversification	Species-rich regions have intrinsic features that foster the accumulation of species (e.g. climatic regime, seasonality, montane zonation) by promoting regional speciation, suppressing the risk of extinction, or both.	Present-day diversification is faster in the species-rich regions. These regions show fast diversification today as they did in the past.	Dobzhansky (1950), Fischer (1960), Rohde (1992), Brown (2014)
Ecological limits	Species-rich regions provide many ecological resources and niches that allow high numbers of species to regionally coexist.	Present-day diversification is close to zero across most regions. Regional speciation tends to be balanced by regional extinction. Regional richness is best explained by regional environment (e.g. climate, environmental energy and productivity).	Simpson (1953), Sepkoski (1981), Rabosky (2013), Storch and Okie (2019)
Interplay of time, diversification and ecological limits	Species-rich regions have been colonized for a long time and accumulated richness at a decreasing rate, due presumably to increasing competition for easily accessible niches and limiting resources, which has suppressed regional diversification over time.	Present-day diversification is faster across species-poor regions. These regions have been colonized recently and host young faunas. Species-rich regions show suppressed diversification coupled with strong signatures of regional competition.	Van Valen (1985), Schluter (2000), Yoder et al. (2010), Morlon et al. (2010), Harmon and Harrison (2015), Schluter (2016)

What has so far prevented the synthesis remains unclear, but it seems that the three explanations are not entirely exclusive and, in some cases, invoke mechanisms that can interact with each other (Van Valen 1985; Schluter 2016; Stroud and Losos 2016; Machac et al. 2018). This suggests promising groundwork for their possible synthesis. Nonetheless, the explanations also conflict in multiple respects, such that it is possible to derive their diagnostic predictions (Table 1), and each has been supported but also challenged by empirical evidence. For example, it is well-documented that regions which have been colonized for a long-time tend to harbor more species, presumably because they have had more time for speciation and the accumulation of richness (Ricklefs 2006; McGuire et al. 2014). Yet, notable exceptions exist (e.g. the newly formed but hyperdiverse Andes) (McGuire et al. 2014). Similarly, species-rich regions are known to harbor clades that diversified rapidly at some point in their history (Dobzhansky 1950; Stebbins 1974; Jablonski et al. 2006). However, toward the present, many of these clades diversify only slowly (Jetz et al. 2012; McGuire et al. 2014; Schluter 2016; Rabosky et al. 2018). Finally, regional resources, energy, niches, and other proxies for the ecological limits tend to correlate closely with regional richness (Schluter 2000; Losos 2010; Rabosky and Glor 2010). Despite the correlations, most regions and clades continue to accumulate species (Morlon et al. 2010; Schluter 2016) and only few seem to have reached the presumed limit on their richness (Cornell 2013). The conflicting evidence suggests that none of the three explanations paints a complete picture on its own and, to fully capture richness dynamics, the mechanisms hypothesized under the different explanations might need to be integrated.

Interactions between the mechanisms remain largely unresolved. But it has been theorized (Simpson 1953; Sepkoski 1981; Van Valen 1985; Stroud and Losos 2016) that regional diversification declines over time, as species gradually accumulate within a region. This process can be modulated by historical changes in regional climate, productivity, and by the expansion or contraction of biomes within which the regions lie (Ricklefs and Schluter 1994; Fine 2015; Schluter 2016). In any case, as regional richness increases, easily accessible niches become filled with species, resource availability declines and so do regional opportunities for ecological speciation, such that regional diversification tends to gradually slow down (Simpson 1953; Schluter 2016; Stroud and Losos 2016). Moreover, the increasingly limiting resources raise the competition pressure within the region, which compresses the population sizes of the resident species and increases their vulnerability to extinction (Van Valen 1985; Storch et al. 2018), which suppresses regional diversification even further (Simpson 1953; Van Valen 1985; Schluter 2009; Yoder et al. 2010; Storch and Okie 2019). This scenario, whose different elements have been commonly invoked in previous work (Simpson 1953; Walker and Valentine 1984; Van Valen 1985; Rabosky 2013; Storch and Okie 2019), produces its own testable predictions (Table 1), namely that species-rich regions have been colonized for a long time and show suppressed diversification, coupled with signatures of competition (Simpson 1953; Van Valen 1985). In contrast, newly colonized, species-poor regions show fast diversification and weak to no signs of competition (Simpson 1953; Van Valen 1985; Schluter 2000; Machac et al. 2018). Currently, empirical support for this scenario remains limited (McPeek 2008; Rabosky 2013; Stroud and Losos 2016; Machac et al. 2018) and, contrary to its diagnostic predictions, species-rich regions are typically expected to show fast, not slow, diversification (Ricklefs 2006; Rolland et al. 2014). Moreover, other scenarios have also been theorized (e.g., diversity begets further diversification) (Benton and Emerson 2007; Erwin 2008; Machac and Graham 2017; Souto-Vilarós et al. 2019). As a result, how the mechanisms hypothesized under the different explanations interact with each other remains unresolved, and we have limited knowledge as to how such interactions, should they occur, might be uniform across taxa and richness gradients.

Two types of gradients are particularly pervasive, regional and elevational, whereby richness declines from the tropical regions toward the temperate (Pianka 1966; Whittaker 1972; Gaston 2000) and from lowlands toward highlands (Rahbek 1995; Graham et al. 2014). Given their similarities, the two types of gradients might result from the same mechanisms, and elevational gradients have often been used as suitable substitutes for studying regional gradients (Whittaker 1972; Stevens 1992). However, some have argued that the similarities are misleading, given that the gradients form over dramatically different scales, both geographic and temporal (Rahbek 2005), such that the mechanisms responsible for their formation might differ significantly in their relative effects and interactions (Rahbek 1995, 2005; Graham et al. 2014). By studying the gradients side by side, including their points of similarity and divergence, we might be able to disentangle the mechanisms and elucidate the formation of richness gradients in general.

Richness gradients are particularly dramatic in the New World. In the Neotropics, the ancient and highly productive rainforests of the Amazon (Hoorn et al. 2010) and the mountain-slopes of the Andes (Hoorn et al. 2010; McGuire et al. 2014) constitute the most species-rich biodiversity hotspots on the planet, harboring the highest regional concentration of plants, amphibians, birds, and mammals (Myers et al. 2000). The confluence of long history, enormous productivity, and historically fast diversification makes the Neotropics, and the greater New World, a well-defined evolutionary arena, uniquely suited to investigate the interplay between the three explanations (Hawkins et al. 2003; Hoorn et al. 2010; Jetz et al. 2012; McGuire et al. 2014).

In this study, I investigate how the effects of time, diversification rates, and ecological limits together shape richness gradients across regions and elevations (Willis 1922; Stebbins 1974; Ricklefs and Schluter 1994; Rosenzweig 1995; Mittelbach et al. 2007). To evaluate the relative effects and the interplay of these explanations, I study five taxa of birds confined to the New World (hummingbirds, tanagers, tyranids, furnariids, and thamnophilids) that together span over 1500 species with highly resolved phylogenies, geographic distributions, and elevational ranges. I find that the explanations are differentially important, but each contributes uniquely to the formation of richness gradients. Importantly, I find that the mechanisms they invoke interact in a remarkably consistent manner across taxa and scales. Knowledge of these interactions may begin to pave the way toward formulating a synthesis as to why some regions and elevations harbor dramatically more species than others.

## Materials and Methods

Focusing on five taxa from the New World, whose respective richness gradients were shaped by the same template of regional geography (e.g., landmass boundaries, configuration of the montane ranges), history (e.g., uplift of the Andes, flooding of the Amazon), and biotic background (e.g., the Great American Interchange, megafaunal extinctions) (Hoorn et al. 2010), profitably narrows down the range of confounding factors. Despite having evolved within the same region, the five taxa differ significantly in their biology. While hummingbirds are highly-specialized nectarivores, physiologically constrained by their rapid metabolism that allows them to function at high elevations, tyranids span many generalist species that vary greatly in their diet and body size. Some of the analyzed taxa are known for species with idiosyncratic foraging strategies (many of the ant-following thamnophilids) and nesting behavior (mud-nests in furnariids). I refrained from analyzing all birds, given that the avian phylogeny has lately been in flux (Jetz et al. 2012; Jarvis et al. 2014; Hedges et al. 2015; Prum et al. 2015), and the effects of unresolved phylogenetic relationships would be further exacerbated by the dramatic differences in the quality of the geographic and elevational data available for different parts of the avian phylogeny (IOC World Bird List v8.1, IUCN 2018). Consequently, many of my results would be hard to interpret, owing to hidden biases and errors, which motivated me to focus only on taxa for which high-quality data are available, can be compiled from and compared across multiple sources (Derryberry et al. 2011; McGuire et al. 2014; Quintero and Jetz 2018), which allows for a straightforward validation of my results. Working with multiple taxa further allowed me to search for robust trends that have emerged repeatedly and largely independently within the same well-defined region despite the significant differences in the taxa’s life-histories.

My analyses were implemented across regions and elevations. Regions were defined as $1 \times 1$ degree grid cells (Hurlbert and Jetz 2007) and biomes (Supplementary Fig. S43 available on Dryad at https://dx.doi.org/10.5061/dryad.b5mkkwh96) (Olson et al. 2001). Elevational results were compiled across 100 m-wide elevational bands (150 m and 200 m cutoffs produced similar trends). Because grid cells, biomes, and elevations cover different scales, they may diverge in some respects while revealing cross-system commonalities. Similarly, montane systems differ in their climate, seasonality, and topography. Although investigating these differences could be interesting in its own right, I argue that we largely lack a synthetic perspective that would abstract from the differences in order to identify robust common trends, needed to integrate the currently conflicting results and hypotheses (Table 1) (Mittelbach et al. 2007; Wiens 2011; Cornell 2013; Graham et al. 2014). Consequently, it is not my objective to examine and describe the results for each of the montane systems, biomes, and elevations. Instead, I search for the trends. Their knowledge might guide further research and more detailed investigation in the future (e.g., by identifying regions and taxa that defy the trends).

### Phylogenies, Regions, and Elevations

Phylogenetic data were taken from multiple sources for each of the studied taxa (Supplementary Table S1 available on Dryad) (Derryberry et al. 2011; Jetz et al. 2012; McGuire et al. 2014; Hedges et al. 2015). I confirmed that different source phylogenies produced mutually consistent estimates of species-level (Supplementary Figs. S1–S10 available on Dryad) and regional-level diversification (Supplementary Figs. S11–S15 available on Dryad) that led to the same conclusions (Supplementary Figs. S1–S15 available on Dryad). The phylogenies (Derryberry et al. 2011; Jetz et al. 2012; McGuire et al. 2014; Hedges et al. 2015) combined information from multiple segments of the avian genome with time-calibration derived from the fossil record, combined with molecular clock, as well as previously constructed trees and backbones (Supplementary Table S1 available on Dryad). Species names within the examined phylogenies were aligned with the currently recognized authoritative species lists under IOC World Bird List v8.1. To account for missing species (species not included in the phylogenies, or included but without molecular sequences), three different strategies were employed, depending on the source phylogeny. Specifically, (i) trees with species randomly imputed into the phylogeny were combined into a maximum clade credibility tree (Jetz and Fine 2012), (ii) missing species were imputed into the phylogeny, based on known taxonomy, by previous authors (Hedges et al. 2015), (iii) missing species were accounted for statistically within the diversification analysis (Rabosky 2014). The three strategies produced virtually identical results, and so did the different source phylogenies for the five taxa (Derryberry et al. 2011; Jetz et al. 2012; McGuire et al. 2014; Hedges et al. 2015), suggesting that my results are sufficiently robust to warrant meaningful conclusions (Supplementary Figs. S1–S15 available on Dryad). For details on the phylogenies and diversification results, see the Supplementary material available on Dryad.

Geographic distributions were taken from IUCN (International Union for Conservation of Nature, 2018) and analyzed across grid cells and biomes. Breaking species distributions into grid cells of appropriate equal sizes ($1 \times 1$ degree for vertebrates) has been shown to limit false-presence errors and target the geographic scale at which birds presumably perceive their environment (Rahbek 2005; Hurlbert and Jetz 2007). But grid cells have also been criticized, as they are rarely statistically independent, the grid-cell patterns might be driven by wide-ranging species (Jetz and Rahbek 2002; Quintero and Jetz 2018), and because in situ speciation in birds typically unfolds over areas larger than grid cells (but see Fjeldsa et al. 2012; Jetz et al. 2012). Biome-level analyses circumvent these issues, but produce their own problems, such as the failure to capture variation within biomes and the low statistical power, resulting from the small number of some types of biomes in the New World (Jetz and Fine 2012; Fine 2015). For these reasons, I used both grid cells and biomes for my analyses. Since both returned principally similar results, I primarily report the grid-cell results, which provide more detailed insight. But biome-based results are summarized below (see Results and Discussion section) and detailed in the Supplementary Material available on Dryad (Supplementary Figs. S44–S49). The definition of biomes followed Olson et al. (2001), as depicted and detailed in the Supplementary Material (Supplementary Fig. S43).

Elevational ranges were compiled from two sources (Karger et al. 2017; Quintero and Jetz 2018). First, the information on the minimum and maximum elevation for each species was taken from the recently published curated database of Quintero and Jetz (2018). Second, the same information was derived from the geographic maps of species distributions, using the global digital elevation model within Chelsa (Karger et al. 2017). Because the latter source is arguably cruder than the former, it was used to confirm the robustness of my elevational results. The results were further tested for sensitivity toward outlier values arising typically at the edge of the gradient (harboring less than 3 or 5 species), and these values were omitted from the analysis if they qualitatively changed the broader trend that typified most of the gradient. Elevational data are notoriously prone to inaccuracy, and validating my results across alternative data sources, with and without possible outliers, raised the robustness of the elevational results.

### Estimating the Time for Speciation

Under time-based explanations, regional richness depends on the time that the regional fauna has had to accumulate species (Table 1) (Willis 1922; Stephens and Wiens 2003). Regional faunas that are rich in species should therefore be relatively old (Wiens et al. 2010; Hutter et al. 2013; Marin and Hedges 2016; Oliveira et al. 2016; Economo et al. 2018; Marin et al. 2018). Previous simulations and empirical work (Oliveira et al. 2016; Economo et al. 2018; Marin et al. 2018) demonstrated that the age of regional fauna can be realistically captured by the mean phylogenetic distance (MPD) between the species that reside within the region. MPD is also robust toward outlier species (e.g., unrelated species newly introduced into the regional community) and toward regional richness (Oliveira et al. 2016). MPD was calculated for each of the analyzed regions (grid cells and biomes), using the R package *picante* (Kembel et al. 2010), and served as a measure of the regional fauna’s age.

Null modeling was used to ensure that only biologically informative MPD values were used for downstream analyses. Specifically, MPD values were calculated for null communities (functions *richness, sample.pool, phylogeny.pool, trialswap* under the *picante* function *ses.mpd*) assembled by randomly selecting species (from the sample pool, from the phylogeny pool, or through randomizing the community matrix), while holding regional richness constant (Gotelli 2000; Kembel et al. 2010). MPD values that fell within the null expectations were removed from analysis, as they belonged to faunas whose inferred age might have been largely predetermined by their richness (Gotelli 2000). The results across different null models were compared to confirm that the procedure used to estimate age has no effect on the main conclusions.

The relative times were further confirmed against ancestral reconstructions. The reconstructions confirmed that the five taxa originated in tropical climates while temperate climates were colonized only later and therefore have had less time to accumulate richness. Ancestral reconstructions were implemented for the key dimensions of the climatic niche in birds: the general climate within the region (PC1, PC2), mean annual temperature (Bio1), annual precipitation (Bio12), and environmental productivity (AET, NPP) (see the Supplementary material available on Dryad). The reconstructions were implemented only for the most phylogenetically conserved dimensions of the climatic niche (Bio1, AET) after testing for phylogenetic signal, using Pagel’s lambda (Pagel 1999) and Blomberg’s K (Blomberg et al. 2003), implemented in the R packages *ape* and *picante* (Paradis et al. 2004; Kembel et al. 2010). I refrained from directly reconstructing the dispersal within the five taxa from one region to another because such reconstructions are currently feasible only for dozens of species and regions (Ronquist and Sanmartin 2011; Matzke 2014) and become computationally intractable and statistically problematic as the size of the data increases (as in my case involving 32 biomes, over 2500 grid cells, and 1530 species) (Goldberg et al. 2011; Rabosky and Goldberg 2015). Ancestral reconstructions were confirmed against known aspects of historical biogeography and dispersal within the studied taxa (Ericson et al. 2003; Ericson 2011; McGuire et al. 2014; Cracraft and Claramunt 2017).

### Estimating Diversification Rates

Regional diversification captures the rate at which species accumulate within a region. It was calculated by averaging present-day diversification rates of species residing within each of the studied regions (using arithmetic mean, harmonic mean, and the median, which returned consistent results).

Regional patterns in present-day diversification may help distinguish between the three explanations for richness gradients (Table 1). Under time-based explanations, diversification proceeds in a clock-like manner, such that some regions are more species-rich than others simply because they have had more time to accumulate species. Consequently, present-day diversification is predicted to be uniform across regions and elevations (Willis 1922; Stephens and Wiens 2003). Under diversification-based explanations, some regions harbor more species than others because their intrinsic features (e.g., environmental heterogeneity, dispersal barriers, topography) foster speciation, suppress extinction, or both. Consequently, present-day diversification is predicted to follow richness gradients and therefore decline from the tropics toward the temperate and, correspondingly, from lowlands toward highlands (Dobzhansky 1950; Fischer 1960; Jablonski et al. 2006). Under the explanations invoking ecological limits, species-rich regions afford more energy and resources that allow more species to regionally coexist; regional richness is stabilized at an equilibrium value set by environmental conditions, and regional speciation is balanced by regional extinction. Consequently, present-day diversification is predicted to be close to zero across all regions and elevations (Sepkoski 1981; Mittelbach et al. 2007; Rabosky 2013). Additionally, under the scenario postulating that diversification proceeds fast in the newly colonized regions but gradually decelerates as regional richness increases and species begin to compete for regional resources, present-day diversification is predicted to increase from the tropics toward the temperate and, correspondingly, from lowlands toward highlands (Table 1) (Simpson 1953; Cornell 2013; Machac et al. 2018; Storch and Okie 2019). Based on these diagnostic predictions, it should be possible to distinguish between the explanations invoking time, diversification rates, ecological limits, but also the integrative scenario that postulates an interplay of these mechanisms (Table 1).

Two methods were used to estimate present-day diversification: DR and BAMM (Jetz et al. 2012; Rabosky 2014). DR makes minimal assumptions about the diversification process, which is assumed to be time-homogeneous and producing an exponential growth in species richness. BAMM is biologically more realistic, accommodates time-heterogeneity, and permits exponential growth but also slowdowns and accelerations in diversification rates. However, some of BAMM’s premises have been questioned (Moore et al. 2016; Rabosky et al. 2017). Since DR and BAMM differ principally in their underlying assumptions and limitations, they are unlikely to converge on similar results, unless the results are firmly grounded in the structure of the phylogenetic data and warrant robust conclusions.

DR, defined as the inverse of the evolutionary distinctiveness (Isaac et al. 2007; Jetz et al. 2012), was calculated in the R package *picante* (Kembel et al. 2010). BAMM (Bayesian analysis of macroevolutionary mixtures) (Rabosky 2014) was implemented under five Markov chain Monte Carlo chains that were run for 10 million generations with the sampling frequency of 1000 generations. To circumvent issues with priors selection, the priors for speciation and extinction rates were set on the values expected under the homogeneous birth–death process. To confirm convergence across chains, I estimated the effective sample sizes for the number of regime shifts and for the rate parameters, ensuring they exceeded the recommended threshold of 500 (Rabosky 2014). Consequently, I obtained posterior distributions for the key parameters (speciation, extinction) needed to estimate species-level diversification rates. Importantly, for each of the five taxa, I evaluated the correlation between BAMM rates and DR rates, using Spearman’s rank correlation. In addition, I confirmed that BAMM and DR converged on similar patterns of regional diversification.

### Estimating the Ecological Limits

Regional richness has been hypothesized to depend on the total amount of energy and resources within the region (Table 1). Energy and resources are difficult to quantify directly but have been demonstrated to correlate with regional climate and productivity (Hawkins et al. 2003; Šímová and Storch 2016). Regional climate was characterized, using 19 bioclimatic variables from Chelsa (Karger et al. 2017). To avoid collinearity issues, I used variables previously identified as most relevant to avian biogeography, mean annual temperature (Bio1) and annual precipitation (Bio12) (Hawkins et al. 2003). In addition, I combined all of the bioclimatic variables into two composite variables (PC1, PC2) representing regional climate, using principal component analysis (PCA). PC1 and PC2 captured the general climate within the region, blending information on temperature, precipitation, and seasonality, while explaining 76.63% of the variance in the climatic data (PC1 factor loadings: Bio1 $=$ 0.918, Bio12 $=$ 0.734, Bio4 $=-0.890$; PC2 factor loadings: Bio1 $= -0.373$, Bio12 $=$ 0.634, Bio4 $=$ 0.004). Detailed results of the PCA, including eigenvalues and factor loadings for all climatic variables, are given in Supplementary Table S2. Regional energy and productivity were approximated as actual evapotranspiration (AET) and net primary production (NPP). AET and NPP data were taken, respectively, from the MODIS Global Evapotranspiration Project (MOD16) (Mu et al. 2011) and MODIS GPP/NPP Project (MOD17) (Zhao et al. 2005).

### Relative Effects and the Partitioning of Variation

After the three classes of predictors were compiled (representing time, diversification rates, and ecological limits), I evaluated their relative effects, using regressions and variation partitioning. These analyses were implemented within the framework of generalized linear models with a gamma link function that flexibly accounts for heteroscedasticity (Nelder and Wedderburn 1972), a feature that was necessary to accommodate the inflated residual variation typifying regions with low richness. Regional richness was regressed against the predictors representing time (MPD calculated under the four types of null models), diversification rates (arithmetic mean, median, and harmonic mean, based on DR and BAMM), and ecological limits (Bio1, Bio12, PC1, PC2, AET, NPP). To ensure meaningful comparisons, the number of predictors was chosen to be balanced across the three classes of variables. MPD predictors were more collinear than those representing diversification and ecological limits, but similar results were recovered when some of the predictors were changed or removed. Variation partitioning was used to uncover the effects of each predictor class while accounting for the effects of the other two classes. An alternative approach to variation partitioning would be to estimate the standardized effect sizes for each predictor class ($\beta _{\rm st})$. But this approach requires full data (with no missing values) across the full set of combinations of the predictors, which reduces the size of the data set significantly (to 30% of the original data set) and therefore inadvertently leads to the loss of potentially important biological information.

Regression analyses were implemented with and without statistically correcting for spatial autocorrelation (Diniz-Filho et al. 2003). The corrections have been argued to distort geographic data in nontransparent ways and deemed unnecessary under some circumstances (e.g., when the data are sampled across a regular grid, as in my case) (Diniz-Filho et al. 2003); still, it seems important to account for the fact that the analyzed grid cells are rarely independent from each other, given that adjacent cells encompass similar species and environments. Moreover, the lack of correction would place equal emphasis on the small-ranging and wide-ranging species, but because the latter occur across many more grid cells than the former, wide-ranging species would have an effectively greater influence on my results. To mitigate these issues, I reran my analyses using the generalized least squares where spatial structure was captured by the variance–covariance matrix derived from the geographic distances among the analyzed grid cells. Spatial covariance was modeled with the nugget effect under four parametric functions: linear, exponential, spherical, and Gaussian. The best-fitting covariance function was identified using Akaike’s information criterion, and its corresponding semivariograms were examined to statistically confirm that the identified function captured the spatial autocorrelation satisfactorily. As a supportive measure, I confirmed my results across biomes. Unlike grid cells, biomes represent largely independent units for geographic analyses, with mutually independent faunas and evolutionary histories, such that their analyses do not require spatial corrections (Jetz and Fine 2012; Belmaker and Jetz 2015). Biome-based results therefore provided a supplementary validation of the grid-cell results, which were calculated with and without the spatial corrections.

### The Interplay of the Three Explanations

Previous theory suggests a scenario that might integrate the mechanisms invoked under each of the three explanations (Simpson 1953; Van Valen 1985; Schluter 2016; Machac et al. 2018; Storch and Okie 2019). Specifically, regional diversification has been proposed to decline over time, as the number of species within a region increases and becomes increasingly limited by competition for regional resources (Simpson 1953; Van Valen 1985; Brown 2014). Even though competition is notoriously hard to demonstrate outside experimental studies (Webb 2000; Webb et al. 2002; Cavender-Bares et al. 2009; Graham et al. 2009), it has been argued to leave diagnostic signatures in the phylogenetic structure of regional communities (Webb et al. 2002). Under the classic principle of competitive exclusion whereby closely related species with similar niche requirements cannot stably coexist (Darwin 1859; Hutchinson 1957; Mayfield and Levine 2010), competition produces communities whose species are less related to each other than would be expected by chance (Webb 2000; Webb et al. 2002). Relatedness, measured in terms of the net relatedness index (NRI), may sometimes be confounded by factors independent of competition (Cavender-Bares et al. 2009), and recent work has problematized the connection between relatedness and competitive exclusion (Mayfield and Levine 2010). For these reasons, NRI cannot serve as definitive proof of competition within any particular community. However, some insight might be gained by searching for systematic trends in NRI across a collection of communities positioned along a richness gradient and by comparing such trends with predictions derived from previous theory (Cavender-Bares et al. 2009; Graham et al. 2009; Machac et al. 2011) under the assumption that competition tends to be stronger among close relatives (Darwin 1859; Hutchinson 1957; Webb et al. 2002; Cavender-Bares et al. 2009).

Building on theory (Simpson 1953; Van Valen 1985; Rabosky 2013; Machac et al. 2018), I test the prediction that species relatedness (NRI) decreases across the gradient of communities from the temperate toward the tropics (Dobzhansky 1950; Schemske et al. 2009) and from highlands toward lowlands (Graham et al. 2009), as expected if competition tended to be stronger within the species-rich regions (Simpson 1953; Van Valen 1985; Yoder et al. 2010; Machac et al. 2018; Storch et al. 2018). I further test the prediction that these changes are coupled with a decrease in regional diversification, as expected if competition suppressed diversification rates (Van Valen 1985; Rabosky 2013; Simpson 1953). Rejecting these predictions might encourage revisiting the theory. Supporting the predictions might motivate further detailed investigation. In any case, my study would be incomplete without an, at least tentative, attempt to test the predicted cross-community trends in the proxies for competition, which has been theorized to represent the mechanistic link between the changes in regional richness and regional diversification. Nonetheless, NRI results need to be interpreted with caution and with the above-stated caveats in mind.

Moreover, the predicted correlations between regional diversification and NRI might result, at least in principle, for purely statistical reasons, given that both variables are derived from the phylogeny. Even though such statistical effects seem unlikely to be pronounced, as discussed in the Supplementary material, I evaluated their magnitude, using null models. The null models were defined in line with the standard practices in the field (Gotelli and Graves 1996; Gotelli 2000), so they would preserve the phylogeny, the number of species occurring within each region, and the number of regions that each species occupies (Gotelli and Graves 1996). This was achieved by reshuffling species names along the occurrence matrix (species $\times$ regions), thus preserving the row and column sums of the matrix, and changing only the degree to which related species tended to occupy the same region or avoid each other (Gotelli and Graves 1996; Gotelli 2000; Kembel et al. 2010). The reshuffling was repeated 100 times for each of the five taxa (hummingbirds, tanagers, tyranids, furnariids, and thamnophilids), and the resultant null correlations were compared with the empirical ones. The reasoning behind the choice of the null models and their execution is detailed in the Supplementary material available on Dryad.

### Sensitivity of the Results

My analyses were repeated separately for each of the five taxa (hummingbirds, tanagers, tyranids, furnariids, and thamnophilids) under multiple statistical setups that involved two to three phylogenies analyzed for each taxon (Graham et al. 2009; Derryberry et al. 2011; Jetz et al. 2012; Hedges et al. 2015), using two principally different diversification methods (BAMM, DR), four types of null models used to estimate time (*richness, sample.pool, phylogeny.pool*, *trialswap*), multiple measures of climate (Bio1, Bio12, PC1, PC2) and productivity (AET, NPP), and elevational data from two alternative sources (Karger et al. 2017; Quintero and Jetz 2018). My analyses were repeated for biomes (Olson et al. 2001), grid cells (1x1 degree), and elevations (100 m bands), and some of the main results (correlations between regional diversification and NRI) were confirmed against null models (Gotelli and Graves 1996; Kembel et al. 2010). Moreover, I confirmed that the taxa produced similar results largely independently of their shared ancestry by testing for phylogenetic signal in my main results, using Blomberg’s K and Pagel’s lambda (Pagel 1999; Blomberg et al. 2003; Revell 2011), as detailed in the Supplementary material. These measures were devised to ensure that my conclusions would be robust and largely independent of the choice of the data and the methods of their analysis.

## Results

**Figure 1.**
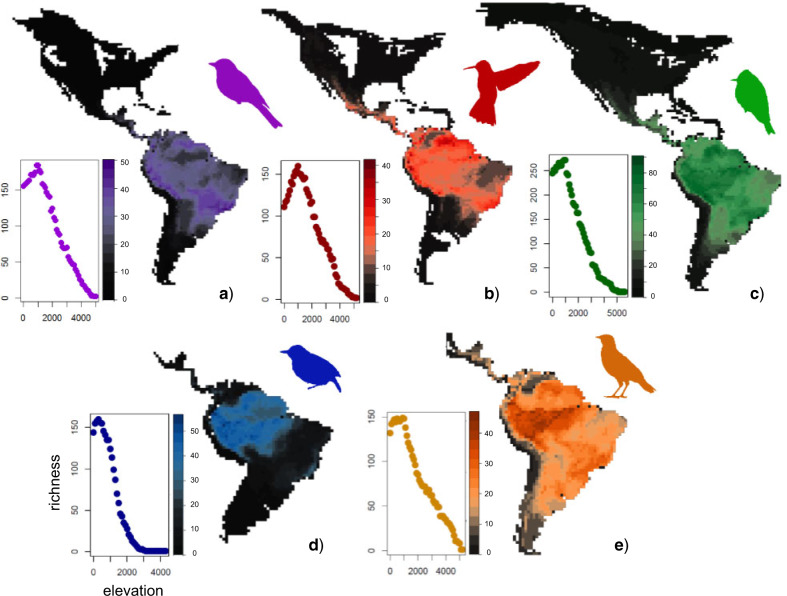
Bird richness across regions and elevations of the New World. Most species are concentrated in the tropics and in the lowlands. Richness declines toward the temperate and toward the highlands, in each of the five families: a) tanagers (Thraupidae), b) hummingbirds (Trochilidae), c) tyrant flycatchers (Tyrannidae), d) antbirds (Thamnophilidae), and e) ovenbirds (Furnariidae). Elevational panels indicate the global richness of the family at given elevation (in meters above the sea level).

My results confirmed that each of the five taxa shows marked richness gradients. Specifically, species richness declined from the tropics toward the temperate and from lowlands toward highlands (Fig. 1). I further found that diversification slowed down over time in each of the five taxa and, by extension, within each of the regions that these taxa occupy (Supplementary Figs. S1–S10). Despite the slowdowns, diversification did not halt completely, declined to only $ \approx $ 50% of its maximum value, and therefore seems to produce further richness across grid cells, biomes, and elevations (Fig. 4, Supplementary Figs. S11–S15). Ancestral reconstructions confirmed that the taxa originated in the warm and highly productive climates, typifying lowlands, especially in the tropics, and only later colonized the cooler and less productive temperate and highland climates (Supplementary Table S3, Supplementary Figs. S16–S25), a result consistent with the prevailing natural-history knowledge (Ericson et al. 2003; Ericson 2011; McGuire et al. 2014; Cracraft and Claramunt 2017).

**Figure 2.**
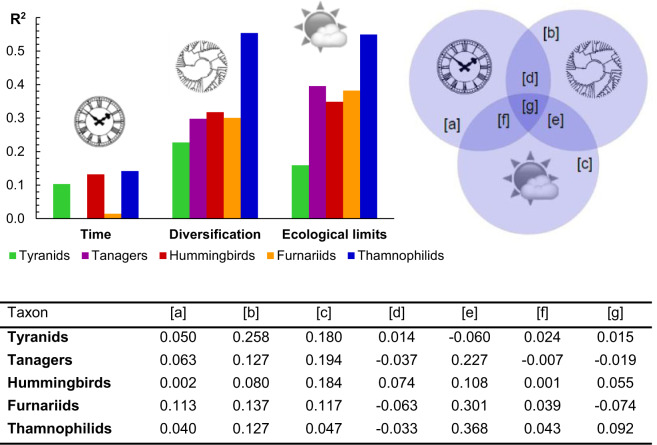
Partitioning the variation in regional richness. The relative effects of time, diversification rates, and ecological limits (top panel) indicate that regional richness is best explained by the effects of climate and productivity ($R^{2} \approx 0.40, P < 0.001$). However, detailed results (bottom panel) reveal that the three classes of effects overlap significantly, and each of them makes a unique contribution. The notation in the diagram (top right) corresponds with table columns (bottom), detailing the variation explained by the different combinations of effects.

**Figure 3.**
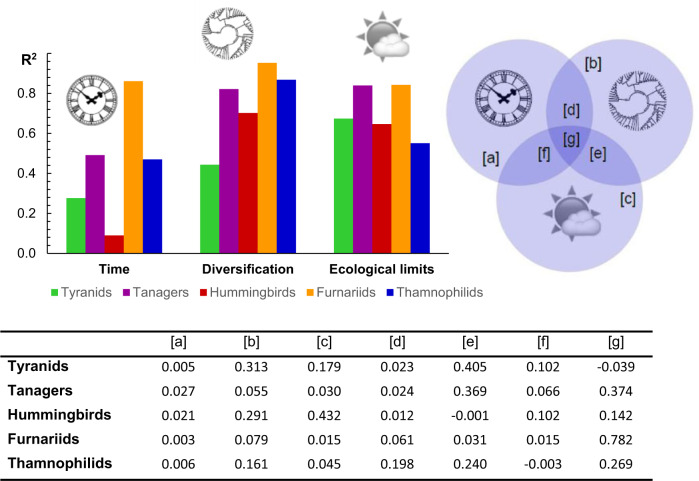
Partitioning the variation in elevational richness. The relative effects of time, diversification rates, and ecological limits (top panel) indicate that elevational changes in richness are best explained by climate and productivity ($R^{2} \approx 0.70, P < 0.001$). Detailed results, however (bottom panel), reveal that the three classes of explanations overlap significantly in their effects. The notation in the diagram (top right) corresponds with table columns (bottom), detailing the variation explained by the different combinations of effects.

Regressions revealed that each of the three classes of predictors (time, diversification rates, ecological limits) contributes significantly to the differences in species richness across grid cells. Ecological limits, expressed as regional climate (Bio1, Bio1, PC1, PC2) and productivity (AET, NPP), explained most of the variance in grid cell richness across the five taxa ($R^{2} \approx 0.40, P < 0.001$) (Fig. 2). Diversification rates were less influential ($R^{2} \approx 0.30, P < 0.001$), and the smallest effect, though still significant, was that of time ($R^{2} \approx  0.10, P < 0.001$) (Fig. 2). The same relative contributions of ecological limits, diversification rates, and time were uncovered for biomes (limits: $R^{2} \approx 0.90, P < 0.001$, diversification: $R^{2} \approx  0.70, P < 0.001$, time: $R^{2} \approx 0.50, P < 0.001$) (Supplementary Fig. S44) and elevations (limits: $R^{2}  \approx 0.70, P < 0.001$, diversification: $R^{2} \approx 0.60, P < 0.001$, time: $R^{2} \approx 0.20, P < 0.001$) (Fig. 3). The results were further corroborated by the variation partitioning which, unlike the regressions, estimates the effects for each of the three classes of predictors while accounting for the effects of the other two classes (Figs. 2 and 3). These results uncovered substantial overlaps between the examined effects (Figs. 2 and 3), suggesting possible interactions between the mechanisms involving time, diversification rates, and ecological limits (see below).

**Figure 4.**
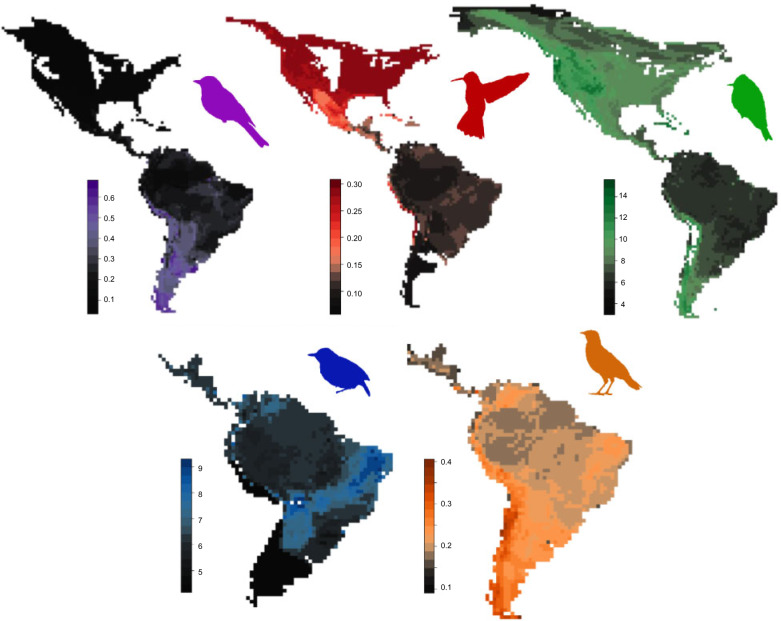
Regional diversification. Present-day diversification tends to be fast in regions with low richness (esp. in the temperate) and slow across the species-rich regions (esp. in the tropics). The pattern is consistent across the five taxa examined. Taxon-specific silhouettes and color-coding correspond with the preceding figures. Maps indicate mean diversification rate of the species occurring across regions ($1 \times 1$ degree grid cells) covering the New World.

Surprisingly, none of the predictions derived from the three explanations regarding present-day diversification were supported (Table 1). Instead, I found that present-day diversification was particularly fast across species-poor regions (esp. the Nearctic) and slow across species-rich regions (esp. the Neotropics) (Fig. 4). This pattern was particularly robust. The negative correlation between present-day diversification and richness held across each of the five taxa at the level of grid cells ($R^{2} \approx 0.20, P < 0.001$) (Supplementary Figs. S33–S37) and biomes ($R^{2} \approx  0.45, P < 0.05$) (Supplementary Figs. S45–S49), with the exception of thamnophilids. Thamnophilids showed a significant negative correlation at the grid-cell level ($R^{2}= 0.216, P < 0.001$) (Supplementary Fig. S35), but not at the biome level ($R^{2} = 0.573, P = 0.246$) (Supplementary Fig. S47), presumably because they are found in four biomes only, such that biome-level analyses had limited statistical power to detect the correlation. Analogous results emerged across elevations ($R^{2} \approx 0.60, P < 0.001$) (Fig. 6, Supplementary Figs. S38–S42), whereby species-poor elevations (e.g., the Andean highlands) showed faster diversification than species-rich elevations (e.g., the Amazonian lowlands). The elevational results held for each of the five taxa (Fig. 6), except hummingbirds. In hummingbirds, different relationships were supported in North and South America (Supplementary Figs. S31 and S32). In the South American Andes, where extant hummingbirds most likely originated, diversification is statistically independent of elevation. In the newly colonized Sierra Madres of North America, however, hummingbird diversification increases from lowlands toward highlands (Supplementary Figs. S31 and S32), which is consistent with the results for the other taxa (Supplementary Figs. S38–S42). These results together indicate that diversification is faster across regions that have been colonized relatively recently, have temperate and highland climates, and are still relatively species-poor (Supplementary Table S3, Supplementary Figs. S16–S25), in line with the integrative scenario indicated in Table 1.

**Figure 5.**
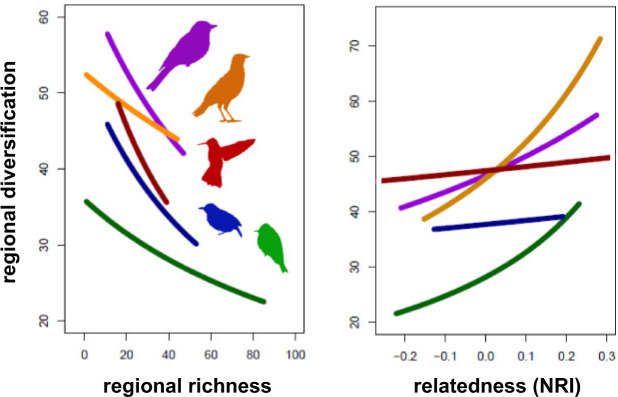
Regional diversification, richness, and possible competition. In each of the five taxa, regional diversification declines with regional richness (left panel). Moreover, regional diversification is suppressed in regions that host unrelated species (NRI $<<$ 0), as expected under increased regional competition (right panel). Taxon-specific silhouettes and color-coding correspond with the preceding figures. The vertical axis indicates relative differences in regional diversification. Full results, including the absolute differences, regression lines, data points, and summary statistics for each taxon separately, are provided in the Supplementary Figures S33–S37 available on Dryad.

**Figure 6.**
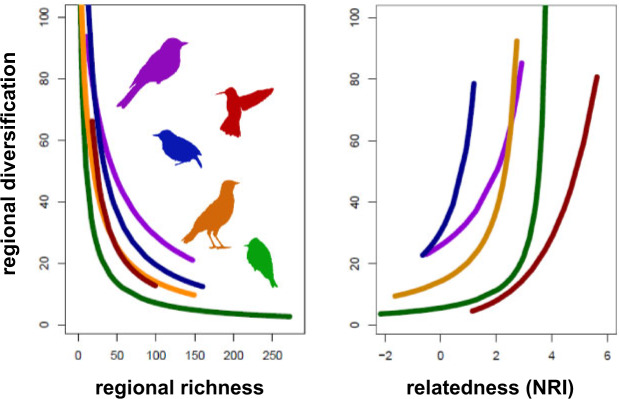
Diversification, richness, and possible competition across elevations. Species-poor highlands show fast diversification while species-rich lowlands show suppressed diversification (left panel). Moreover, suppressed diversification is coupled with strong signatures of possible competition within lowlands (NRI $<<$ 0) (right panel). Taxon-specific silhouettes and color-coding correspond with the preceding figures. The vertical axis indicates relative differences in diversification. Full results, including the absolute differences, regression lines, data points, and summary statistics for each taxon separately, are provided in the Supplementary Figures S38–S42 available on Dryad.

**Figure 7.**
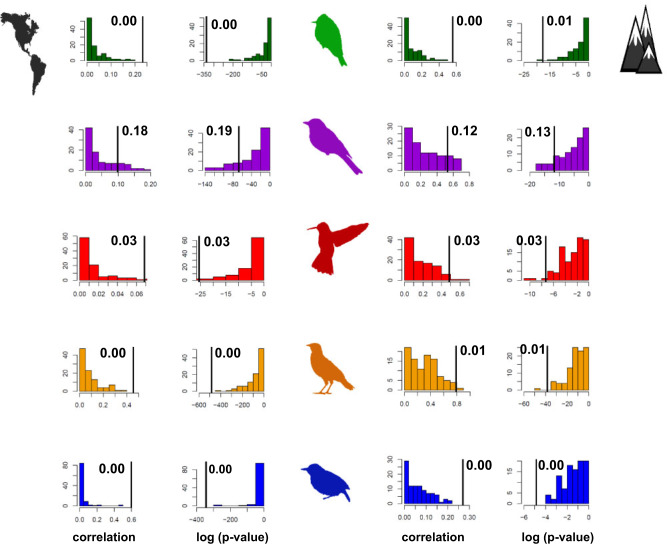
Null models for the correlation between diversification rates and NRI. The results confirm that the empirical correlation (Figs. 5 and 6) cannot be explained solely by the structure of the underlying data (i.e., the phylogeny, the number of species occurring within each region, and the number of regions that each species occupies). Empirical results (vertical lines) are compared against the null results for each taxon separately across regions (panels on the left) and elevations (panels on the right). Taxon-specific silhouettes and color-coding correspond with the preceding figures. Vertical axis indicates the frequency with which the different null results were observed.

The predicted correlation between regional diversification and NRI ($R^{2}  \approx  0.30, P < 0.001$) held across grid cells (Supplementary Figs. S33–S37), biomes (Supplementary Figs. S45–S49), and elevations (Supplementary Figs. S38–S42), corroborating that regions whose resident species are less related to each other than would expected by chance, due possibly to competition, show suppressed diversification. Each of the five taxa supported the same result (details on each taxon are given in Supplementary Figs. S33–S42, S45–S49), with the exception of biome-level results for thamnophilids that were again statistically nonsignificant (Supplementary Fig. S47). Null models confirmed that the empirical correlations cannot be fully explained by statistical effects because they were significantly stronger than expected under the null model (Fig. 7).

Despite being consistent in terms of the main trends, the results for different taxa, grid cells, biomes, and elevations systematically diverged in several respects. The total amount of explained variance tended to be higher for biomes than grid cells, presumably because biomes are associated with lower sample sizes ($N = 32$ biomes) than grid cells ($N \approx 2500$ grid cells, depending on the taxon), which also might have contributed to the nonsignificant results for thamnophilids at the biome-level (above). Still, biomes and grid cells revealed largely congruent geographic patterns (Supplementary Figs. S33–S37, S45–S49), even though grid-cell results uncovered significant variation within some biomes (esp. the Amazon, Cerrados, and North American deserts) (Fig. 4). Similarly, spatial corrections deflated the explained variance in the geographic patterns ($R^{2}  \approx 0.20$) (Supplementary Tables S5–S9), especially in tanagers ($R^{2} < 0.01$). The main results, nonetheless, remained supported even after the spatial corrections (Supplementary Tables S5–S9) and were further confirmed by biome-based results that did not require the corrections (Supplementary Figs. S45–S49). Moreover, the similarities in the main results for the five taxa did not reflect the taxa’s relatedness (Blomberg’s $K \approx 0.90, P > 0.15$; Pagel’s lambda $ \approx  0.05, P > 0.10$) (Table S10), which suggests that the similarity of the results for the individual taxa was not dictated by their shared deep-time history.

Supplementary analyses, which varied the source data and methodology, corroborated the main results. Namely, the phylogenies taken from different sources (Birdtree, TimeTree) (Jetz et al. 2012; Hedges et al. 2015) produced consistent estimates of species-level (Supplementary Figs. S1–S10) and region-level diversification (Supplementary Figs. S11–S15) and so did DR and BAMM (Supplementary Figs. S1–S15). The results on the effects of time showed no substantial differences across the four examined null models (*richness, sample.pool, phylogeny.pool*, *trialswap*) (Supplementary Table S4). Because MPD results, used to evaluate the age of the regional fauna, were confirmed by ancestral reconstructions, they were unlikely to be confounded by regional diversification, which is further corroborated by the fact that regions with low MPD were species-poor (rather than species-rich) (Figs. 1, 4, Supplementary Figs. S16–S25). The same results were supported under elevational data taken from two alternative sources (IUCN 2018; Quintero and Jetz 2018) (Supplementary Figs. S26–S30). Some of my results were sensitive to the choice of the methods and the data, as acknowledged above (e.g., biome-level results for thamnophilids). Because my objective was to identify common trends, I focus my interpretation on the results that proved robust and were supported across taxa, grid cells, biomes, and elevations. Full results, including taxon-specific $R^{2}$, $P$-values, plots, etc., for the above described analyses, are given in the Supplementary material available on Dryad.

## Discussion

My results uncover insights into the dynamics of species richness across regions and elevations. They suggest that richness continues to accumulate everywhere, including the hyperdiverse lowlands of the Amazon and the highlands of the Andes. But the process tends to be faster across those regions and elevations that are species-poor (e.g., Nearctic) and have been colonized only recently (e.g., the Sierra Madres of North America). Conversely, their long-colonized and species-rich counterparts may have undergone fast diversification in the past, but tend to accumulate new species only slowly toward the present (e.g., Amazonian lowlands). The reverse relationship between regional richness and diversification defies the predictions of each of the three prominent explanations for richness gradients (Table 1). Instead, my results (Figs. 4–6) appear consistent with the hypothesis that diversification decelerates over time without necessarily reaching a hard limit on richness (Whittaker 1972; Wiens 2007; Wiens 2011; Schluter 2016). These results held for five taxa of birds (hummingbirds, tanagers, tyranids, furnariids, and thamnophilids) across grid cells, biomes, and elevations (Fig. 4, Supplementary Figs. S1–S15, S33–S42, S45–S49). By illuminating how the mechanisms involving time, diversification rates, and ecological limits interact with each other, my results lay the groundwork for a possible synthesis bridging the three explanations (Rohde 1992; Willis 1922; Simpson 1953; Stebbins 1974; Van Valen 1985; Stephens and Wiens 2003; Mittelbach et al. 2007). Particularly, they reveal the promise of a more dynamic view of richness gradients where the mechanisms hypothesized under the different explanations are investigated together as their interactions unfold over time. They motivate a shift away from the discussion as to which one of the explanations is correct toward a perhaps more promising research concerned with the interplay of the mechanisms and their changing relative importance across regions, taxa, and scales, which eventually gives rise to the dynamics of species richness.

One scenario that would integrate the collection of my results (Willis 1922; Dobzhansky 1950; Simpson 1953; Stebbins 1974; Stephens and Wiens 2003; Ricklefs 2006) posits that regional diversification is negatively diversity-dependent and decelerates as richness increases (Figs. 4–6), presumably because the increasing exploitation of niches and resources limits opportunities for ecological speciation (Sepkoski 1981; Losos 2010; Schluter 2016), raises the competition pressure within the region and compresses the population sizes of the resident species (Van Valen 1985; Storch et al. 2018; Storch and Okie 2019). As the statistical distribution of the population sizes shifts toward smaller populations, regional extinctions from environmental and demographic stochasticity increase and further slow the diversification process down (Simpson 1953; Van Valen 1985; Losos 2010; Storch et al. 2018; Storch and Okie 2019). Despite the slowdown, diversification might not halt completely, as increased competition forces species to diverge along novel axes of their ecological niche (Pianka 1966; Donoghue 2008). Such divergence has been shown to become progressively difficult, once easily accessible niches are filled, but rarely difficult enough to halt diversification entirely (Pianka 1966; Morlon et al. 2010; Harmon and Harrison 2015; Kennedy et al. 2018). Consequently, regional richness increases over time, albeit at continually declining rates, and the longer a region has been colonized, the more species it harbors (Willis 1922; Stephens and Wiens 2003; Marin et al. 2018). This scenario integrates the most robust patterns across my results (Figs. 4–6, Supplementary Figs. S33–S42, S45–S49), and its components find support in previous empirical work (Walker and Valentine 1984; Morlon et al. 2010; Rabosky and Glor 2010; Graham et al. 2018) and theory (Simpson 1953; Stanley 1979; Van Valen 1985). Despite this evidence, other interpretations should not be ruled out and multiple caveats need to be considered.

First, each of the uncovered patterns might be subject to error and inconclusive on its own. Together, however, the patterns display a compelling agreement with the predictions of previous theory (Table 1) (Simpson 1953; Stanley 1979; Van Valen 1985; Losos 2010), and their persistence across different data, setups, and systems (Figs. 2–6) strongly points toward a common underlying narrative. In fact, it seems hard to propose an alternative that would parsimoniously explain the results of the diversification analyses, geographic comparisons, and deviations from null models (Figs. 4–7, Supplementary Figs. S33–S42, S45–S49), without invoking some form of negative diversity-dependence. Second, the use of proxies has its limitations. Proxies for competition (NRI) are particularly contentious, given that they might be confounded by the effects of environmental heterogeneity or dispersal (Cavender-Bares et al. 2009) and given that the link between competition and relatedness, at least at the local scales, has been questioned (Chesson 2000; Mayfield and Levine 2010). For these reasons, I refrain from drawing conclusions about competition within any particular community. Instead, I test for general trends across a collection of communities that would be expected if competition was present and tended to be stronger among relatives, as compared to nonrelated species (Webb 2000; Webb et al. 2002; Cavender-Bares et al. 2009). Less contentious are proxies for time, diversification, and ecological limits. I derived multiple proxies for each to mitigate the risk that some of the three explanations would be captured better than the others. I further relied on multiple null models (e.g., *sample.pool, trialswap* when calculating MPD), statistical methods (e.g., BAMM, DR), and published variables (e.g., AET, NPP), whose choice was guided by authoritative literature in the field (Gotelli and Graves 1996; Evans et al. 2005; Morlon 2014). Time in particular was measured only indirectly, as the age of the fauna within the region, but previous simulations and empirical analyses (Marin and Hedges 2016; Oliveira et al. 2016; Economo et al. 2018) confirmed that the age reasonably approximates the time that the regional fauna has had to accumulate species within a region (Oliveira et al. 2016). Third, even though my results held for five taxa of birds, they might not necessarily hold for other avian or nonavian taxa. Admittedly, four of the five taxa were passerines (Passeriformes: tanagers, tyranids, furnariids, and thamnophilids). But similar results were supported in nonpasserine hummingbirds (Apodiformes) (Supplementary Figs. S31–S32, S37, S42, S49) and, even among the passerines, the relatedness of the taxa did not predict the similarities among their results, suggesting that the similarities arose largely independently of the taxa’s shared evolutionary history (Supplementary Table S10). Nonetheless, further extensions to birds worldwide or nonavian taxa whose richness gradients were shaped by the geographic template of the New World (e.g., mammals, reptiles, amphibians) would be clearly valuable. It would also be illuminating to further refine the variables examined, using more direct measures of competition, time since colonization, and ecological resources. Such measures are notoriously hard to derive, but my results identify which measures might be promising to target in future work.

I found that regional diversification is fast where richness is low (Fig. 4, Supplementary Figs. S33–S42, S45–S49). This pattern was particularly robust, supported by well-established proxies for regional richness and diversification, across each of the five taxa, across grid-cells, biomes, and elevations (Supplementary Figs. S1–S15), and seems compelling in its own right, regardless of its causal links to the rest of my results. Interestingly, the pattern is at odds with the predictions of the prevailing explanations for richness gradients (Table 1). These explanations posit, for example, that species-rich regions have intrinsic features that foster speciation, suppress extinction, or both (Dobzhansky 1950; Stebbins 1974; Stanley 1979; Rohde 1992; Jablonski et al. 2006). Tropical regions, in particular, are presumed to have accumulated high richness because their climatic regime, low seasonality and high stability promote speciation (e.g., high temperatures accelerate biological rates, including life-cycle rates, mutation rates, and speciation rates) (Rohde 1992; Brown 2014) while suppressing extinction (e.g., strong zonation of tropical mountains offers extinction refuges) (Pianka 1966; Janzen 1967; Evans et al. 2005). While it is clear that these intrinsic features of the tropical regions might have facilitated tropical diversification and the accumulation of richness in the past (Janzen 1967; Rohde 1992; Evans et al. 2005; Ricklefs 2006), my results reveal that their effects might be currently overwritten by other factors to the extent that present-day diversification in the tropics does not appear to be fast, but rather slow, and below the levels currently observed in the temperate (Figs. 4–6) (Weir and Schluter 2007; Schluter 2016; Rabosky et al. 2018).

Mine are not the only results for birds that failed to corroborate fast present-day diversification in the tropics (Jetz et al. 2012; Rabosky et al. 2015) even though the evidence for fast tropical diversification, at least in the past, is towering and spans a spectrum of taxa (Jablonski et al. 2006; Ricklefs 2006; Mittelbach et al. 2007; Wiens 2007). Specifically, some studies for birds found no variation in present-day diversification across latitudes (Jetz et al. 2012; Rabosky et al. 2015), possibly, because some of the variation was obscured by the fact that the studies used latitude alone, rather than the entire geographic range of the species (Rabosky et al. 2015), or compared present-day diversification globally, rather than separately for multiple taxa (Jetz et al. 2012). Differences across latitudes were uncovered when comparing the splits between sister-species of birds (Weir and Schluter 2007), which revealed consistently younger splits and therefore faster recent speciation toward high latitudes (Weir and Schluter 2007). Similar results were reported for ray-finned fishes (Rabosky et al. 2018), suggesting that the pattern of fast diversification where richness is currently low might not be limited to birds.

The pattern itself seems to be robust, at least across the taxa where it has been examined, and suggests that even though the tropics presumably acted as “the engine of global diversity” in the past, they might no longer play that role today (Figs. 4–7) (Jablonski et al. 2006; Weir and Schluter 2007; Jetz et al. 2012; Rabosky et al. 2015). What is less clear are the causes of the pattern. Besides the hypothesized negative diversity-dependence (Simpson 1953; Walker and Valentine 1984; Van Valen 1985; Storch and Okie 2019), other explanations seem plausible. For example, tropical biomes covered much of the surface of the Earth and accumulated their enormous richness during the Eocene thermal maximum (Ricklefs and Schluter 1994; Wiens and Donoghue 2004; Fine 2015). The effects of geographic area alone likely accelerated tropical diversification during Eocene by fostering mutation-order speciation, produced by an uneven gene flow over extensive geographic distances and by the resultant accumulation of genetic incompatibilities (Schluter 2009, 2016). Still, these effects were likely outweighed by ecological speciation, produced by local adaptation to the different niches and resources, spread out across the productive and geographically expansive tropical areas (Simpson 1953; Schluter 2000; Losos 2010; Wiens 2011). Given that ecological speciation, which inherently slows down as ecological niches become filled and resources appropriated, seems much more common in nature and produces more richness than mutation-order speciation (Schluter 2009), the effects of historical biome area seem largely compatible with the negative diversity-dependence (Willis 1922; Wiens and Donoghue 2004; Fine 2015). Tropical diversification might have decelerated as tropical biomes contracted toward the equator, providing progressively less geographic area, ecological opportunities, niches, and resources for diversification (Fine 2015; Schluter 2016). Temperate diversification might have followed the opposite trajectory, reaching high rates toward the present, as temperate biomes have been geographically expanding since Oligocene (Ricklefs and Schluter 1994; Wiens and Donoghue 2004; Fine 2015). These effects might have been further compounded by ephemeral speciation in the temperate, where many of the species might be still in the process of formation, given their comparatively younger age. Similar mechanisms have been invoked to explain fast diversification at high elevations, whose environments are typically also newly formed, species-poor, and relatively recently colonized (Quintero and Jetz 2018). Further work is needed to definitively identify the primary cause of the pattern of slow present-day diversification across species-rich regions and elevations (Weir and Schluter 2007; Quintero and Jetz 2018; Rabosky et al. 2018). But some tests could be readily implemented (Losos 2010; Schluter 2016), such as comparing the pattern across clades of different ages and sizes to separate the hypothesized effects of diversity-dependence and geographic area; potentially informative could also be comparisons of ancestral and newly colonized regions, regular and inverse latitudinal diversity gradients (Weir and Schluter 2007; Kozak and Wiens 2012; Graham et al. 2014). Finally, it is hard to refute entirely the possibility that the pattern might be, at least to some extent, influenced by taxonomic practices. While taxonomists supposedly favor finer splitting of the temperate species, such biases seem unlikely to explain the pattern completely, given that it holds for taxa with different histories of taxonomic practice, but also when fitted separately for temperate regions (Supplementary Figs. S31 and S32) and for taxa (such as hummingbirds) whose tropical diversity has been subject to detailed taxonomic research (Bleiweiss 1998; McGuire et al. 2014).

Ecological limits, measured in terms of regional climate and productivity, emerged as the main correlates of regional richness in birds (Figs. 2 and 3, Supplementary Fig. S44). While similar results have been reported before (Hawkins et al. 2012; Jetz et al. 2012; Rabosky et al. 2015), rarely have they been confirmed while explicitly controlling for the confounding effects of time and diversification rates (Pontarp and Wiens 2017; Marin et al. 2018). Even though ecological limits explained the largest share of variation in richness ($ \approx 40{\%}$), time and diversification still made distinctive contributions (Figs. 2 and 3, Supplementary Fig. S44). This suggests that it is not the individual effects of these respective mechanisms, but rather their confluence (Willis 1922; Dobzhansky 1950; Mittelbach et al. 2007), involving an extensive time for the accumulation of species within a region with significant ecological resources and conducive to fast diversification, that produces the most extraordinary hotspots of biodiversity, such as the Amazon or the Andes.

Even though the term “ecological limit” implies a hard cutoff on regional richness (Schluter 2016; Storch and Okie 2019), my results imply that regional resources can suppress diversification without imposing a hard ceiling on the number of species that can co-occur within a region (Wiens 2011; Cornell 2013; Harmon and Harrison 2015). While diversification was relatively slow in the species-rich regions, it was still at $ \approx $ 50% of its maximum value and stayed far above zero (Supplementary Figs. S33–S42), indicating significant potential for further richness growth even within the most species-rich regions of the New World (Fig. 4, Supplementary Figs. S33–S42, S45–S49). This conclusion finds support in diversification results for hundreds of vertebrate, plant, mollusk, and other clades that seem to show diversification slowdowns without any clear indication that their richness would be converging toward an asymptote (Morlon et al. 2010). Phylogenetic and fossil data for multicellular higher taxa, including those within mammals, birds, ferns, or angiosperms, revealed that diversification tends to be slower when evaluated over longer timescales, but rarely approaches zero, corroborating that regional speciation and extinction rarely end up in balance over the long term (Diaz et al. 2019). Considering this evidence, it appears that diversification slowdowns under limiting resources are not necessarily in contradiction with a continued accumulation of species (Wiens 2011; Cornell 2013; Harmon and Harrison 2015; Pontarp and Wiens 2017; Storch and Okie 2019). Consequently, there might be room for integrating explanations that invoke ecological limitation (e.g., limiting resources produce non-asymptotic diversification slowdowns) and time (e.g., richness increases with time for speciation, albeit at potentially declining rates) (Table 1), rather than perceiving the explanations as inherently contradictory.

Besides the main trends, my work uncovers concrete insights for some of the examined regions and taxa. Hummingbirds, in particular, show intriguing results across the montane systems of North and South America (Supplementary Figs. S31 and S32). In the South American Andes, their diversification barely increases with elevation, but the increase becomes steep across the Sierra Madres of North America. My further analyses confirmed that diversification within the South American Andean clades has declined significantly since they colonized the Andes. But the two clades that have only recently colonized North America (the bees and the emeralds) diversify very fast, especially at high elevations of the Sierra Madres. These differences seem to enrich and further corroborate my main findings. Namely, they illustrate that diversification slows down over time, and proceeds at modest rates within the long-colonized, species-rich regions, which have been largely filled with species (e.g., the Andes). In the newly colonized, species-poor regions, conversely, diversification proceeds relatively fast (e.g., the Sierra Madres).

The interplay of mechanisms that unfolds across the entire taxa, such as hummingbirds, may therefore operate in a similar manner also within taxa, such as the bees and the emeralds, and therefore span a range of phylogenetic scales (Supplementary Figs. S9 and S10, S31 and S32). Similarly, the interplay seems to hold at the global scale of latitudinal gradients (North and South America) (Fig. 4), the continental scale of biomes (grasslands, savannahs, broadleaf forests, etc.) (Supplementary Figs. S43–S49), and the regional scale of elevational gradients (the Andes, the Sierra Madres, etc.) (Fig. 6, Supplementary Figs. S38–S42). Because these gradients encompass different phylogenetic, geographic, and temporal scales, they are typically portrayed as only superficially similar (Rahbek 1995, 2005). More research is clearly needed, but my results suggest that richness gradients might be shaped in a more universal fashion, at least in terms of the underlying processes and their interactions (Figs. 5 and 6, Supplementary Figs. S26–S32), than typically believed.

These commonalities notwithstanding, the results for taxa, regions, and scales diverged in several respects. I was generally more successful at explaining richness variation at the biome ($R^{2} \approx 0.70$) than at the grid-cell level ($R^{2} \approx 0.40$) (Fig. 2, Supplementary Fig. S44), presumably because the formation of richness patterns is more deterministic at large scales, where regional fluctuations tend to cancel out (Rahbek and Graves 2001; Hurlbert and Jetz 2007). This can be further compounded by statistical effects, as the lower number of biomes than grid cells produces limited richness variation and lower statistical power (Jetz and Fine 2012), which would explain why the results for some taxa (esp. thamnophilids, which occupy only four biomes) were nonsignificant across biomes but pronounced across grid cells (Supplementary Figs. S35 and S47). Similarly, in tanagers, statistical corrections shifted the correlation between regional diversification and NRI toward nonsignificance (Supplementary Table S5), suggesting that the correlation might be governed by some subclades (such as the seedeaters from genus *Sporophila*) or regions (Patagonia) typified by rapid diversification (Fig. 4, Supplementary Figs. S1 and S2). Hummingbirds also produced divergent results across South and North America (Supplementary Figs. S31 and S32). These cases of divergence are interesting in their own right and could motivate further research into the regions and taxa that depart from the broader trends, which might illuminate when the interplay of the mechanisms that shape richness gradients changes and falls apart.

It is important to acknowledge the sources of error that could have potentially influenced some of my results, including errors associated with the phylogenies, estimates of diversification rates, elevational ranges, estimates of time, and the null modeling. While it is hard to exclude the effects of such errors completely, I have taken multiple precautionary measures to confirm that none of them would overturn my main conclusions. Namely, I repeated diversification analyses for phylogenies from different sources (Derryberry et al. 2011; Jetz et al. 2012; Hedges et al. 2015; McGuire et al. 2014), using different methods (BAMM, DR) (Jetz et al. 2012; Rabosky 2014), and confirmed that the results converged on similar patterns in regional diversification (Supplementary Figs. S11–S15), whereby fast diversification always typified the species-poor regions, and vice versa (Fig. 4). Moreover, I used two types of estimates of species’ elevational ranges, based on the mean elevation of the $1 \times 1$ degree grid cells, lying within a species’ distribution, and based on the recently published global assessment of birds across montane systems (Quintero and Jetz 2018). While the former estimates are much cruder than the latter, both supported similar trends with elevation (Supplementary Figs. S26–S30, S38–S42). The effects of time are notoriously hard to estimate directly (esp. across 100+ regions and species), so I followed the precedent in the field (Oliveira et al. 2016; Economo et al. 2018; Marin et al. 2018) and relied on the age of the regional fauna as a measure of time, confirming that similar results were supported under four different null models (Supplementary Table S4) (Oliveira et al. 2016; Economo et al. 2018; Marin et al. 2018). Further separate null modeling was used to confirm the relationship between regional diversification and NRI (Fig. 7). In this case, the null models confirmed that the detected relationship cannot result merely from the structure of the data (because the models were defined to hold the structure of the phylogeny and the species distributions unchanged) (Fig. 7, see the Supplementary Material on Null models). Finally, my conclusions hold not only across the different precautionary measures detailed above (Supplementary Figs. S1–S15, S26–S30, S38–S42), but also across five taxa of birds with dramatically different life-histories (nectarivorous hummingbirds, generalist tyranids, diet-specialized thamnophilids, etc.). The similarity of the results for different taxa was independent of their relatedness (Supplementary Table S10), suggesting that the taxa produced similar results irrespective of their shared deep-time evolutionary history. Together, these measures corroborate that my main conclusions might be robust and general enough to be detected despite the dietary, nesting, or habitat differences among the analyzed taxa, and despite the differences in the resolution of the phylogenetic and distributional data currently available for these taxa.

## Conclusion

My study revealed the relative effects of time, diversification rates, and ecological limits, while elucidating their possible interplay. Specifically, I found that new species are being accumulated across regions and elevations, but the process seems to unfold faster toward the species-poor edge of richness gradients. This implies that richness gradients might change, fade or erode over time, if all else stayed equal (Weir and Schluter 2007; Mannion et al. 2014; Schluter 2016). Moreover, I found that even though regional climate and productivity correlate closely with regional richness, the correlation does not necessarily imply an ecological limit on the number of species within a region (Figs. 4–6) (Wiens 2011; Cornell 2013; Harmon and Harrison 2015). My results are consistent with several causal narratives, some of which are not mutually exclusive. Multiple robust patterns emerging within my results together suggest negative diversity-dependence, whereby regional diversification declines as regional richness increases over time and species increasingly compete for regional resources (Simpson 1953; Walker and Valentine 1984; Van Valen 1985; Rabosky 2013; Machac et al. 2018). However, these effects could be further reinforced, or partly substituted, by the effects of historical expansions and contractions of the temperate and the tropical biomes, respectively, by ephemeral speciation or taxonomic biases (Ricklefs and Schluter 1994; Jetz and Fine 2012; Fine 2015; Schluter 2016). Uncovering the causality behind the patterns requires further work. But my results set the necessary groundwork and identify promising strategies for such research, such as comparing diversification rates at different time-points in the history of the formation of the gradient (Machac and Graham 2017; Graham et al. 2018). Together, my findings demonstrate how taking a dynamic view of richness gradients, instead of one that is stationary, might bring us closer toward a synthesis of seemingly conflicting results and hypotheses. Consequently, investigating multiple mechanisms together and illuminating how their interplay unfolds over evolutionary time might prove to be a powerful strategy for resolving the enigmas of global biodiversity that inspire biologists ever since Humboldt.

## Supplementary Material

Data available from the Dryad Digital Repository: https://dx.doi.org/10.5061/dryad.b5mkkwh96.
